# An interpersonal nutrition campaign and maternal knowledge and childhood feeding practices: a case study from mothers in rural Indonesia

**DOI:** 10.1186/s13690-020-00444-9

**Published:** 2020-07-09

**Authors:** Cecily Starkweather, Ayla Guarino, Natalie Bennion, Malynne Cottam, Josie McGhie, Kirk A. Dearden, Otte Santika, Hafizah Jusril, Cougar Hall, Benjamin T. Crookston, Mary Linehan, Scott Torres, Cudjoe Bennett, Joshua H. West

**Affiliations:** 1 Department of Public Health, Master of Public Health Program, Brigham Young University, Provo, UT USA; 2 IMA World Health, Washington, DC USA; 3 Reconstra Utama Integra, Jakarta, Indonesia; 4 Center for Health Research, Universitas Indonesia, Depok, Indonesia; 5 Department of Public Health, Brigham Young University, 2139 LSB, Provo, UT 84602 USA; 6 RTI International, Washington, DC USA; 7 Global Health Support Initiative III, Social Solutions Inc, Rockville, USA

**Keywords:** Active feeding, Behavior change, Feeding practices, Indonesia, Interpersonal communication, Stunting

## Abstract

**Background:**

The purpose of this study was to examine the extent to which a national nutrition communication campaign (NNCC) intervention providing interpersonal communication (IPC) was associated with improved knowledge and behaviors related to feeding practices among mothers with children under two years of age in rural Indonesia.

**Methods:**

Data came from a follow-up, cross-sectional survey of 1734 mothers. Key outcomes of interest were minimum meal frequency, minimum dietary diversity and minimum acceptable diet, as defined by the World Health Organization. Associations between exposure to the NNCC intervention and infant and young child feeding (IYCF) knowledge and behaviors were analyzed using adjusted linear and logistic regression, controlling for age, education, and income.

**Results:**

A total of 525 mothers reported exposure to IPC interventions (30.3%). Participation in IPC was associated with increased knowledge of feeding practices (*p* < .0001). Separately, knowledge of feeding practices was related to achieving recommended behavioral practices of minimum meal frequency (*p* = 0.019), dietary diversity (*p* = 0.013), adequate diet (*p* < .001).

**Conclusion:**

These findings underscore the value of increasing maternal knowledge of IYCF practices through IPC interventions as a way to improve behavioral practices and address stunting in rural Indonesia.

## Background

Chronic undernutrition, also known as stunting, is defined as a child being < 2 z-scores at or below the median height-for-age standard [1]. Childhood stunting has life-long consequences and has been linked to increased susceptibility to disease [2], premature death [2], reduced cognitive functioning [3], poor performance in school [4], delays in motor development [4], and reduced productivity in the workplace [5].

Preventing childhood stunting continues to be a major public health challenge. UNICEF’s 2019 *State of World’s Children* reports that globally, at least one in three children under five years of age demonstrates poor growth [6]. In Indonesia, approximately one-third of children 0–59 months are stunted [7]. Findings from the Indonesian Family Life Survey from 2000 to 2014 reveal stunting prevalence increased from 29.7% in 2000 to 32.6% in 2014 among children less than 2 years of age. This increase was significantly associated with a child’s age, pre-lacteal food intake, birth weight, and sanitation practices [8].

Improving ways in which children are fed is one of several strategies for preventing stunting. Results from previous studies provide support for interventions designed to increase maternal knowledge of nutrition and feeding practices to prevent stunting [9–12]. This knowledge includes understanding how to adapt the feeding method to the child’s age and motor skills development, feeding responsively, understanding children’s hunger cues, encouraging the child to eat, recognizing low appetite signs, creating a good feeding environment by reducing distractions, and supervising the child while eating [13].

In developing settings where stunting is most common, numerous barriers exist to adopting these behaviors. These may include a lack of knowledge about best practices, unfavorable cultural norms and beliefs, perceived barriers to change based on responsibilities in the home, and being of lower socioeconomic status [14–16]. A 5-year National Nutrition Communication Campaign (NNCC) aimed at stunting prevention and targeting 10 provinces across the Indonesian archipelago included both mass media communications and interpersonal communication (IPC) approaches. IPC strategies tend to include face-to-face verbal or non-verbal information exchanges, either between two people or in small groups. In particular, IPC interventions included in the NNCC focused on health workers communicating to mothers and caregivers the importance of exclusive breastfeeding for the first 6 months of life, providing appropriate complementary foods according to child’s age, providing a sufficient amount of food, feeding frequently, inclusion of animal-source foods in the diet, and proper hygiene. Channels for the delivery of NNCC IPC messages included maternal health classes and other posyandu (integrated health post) services specific for women and children throughout rural Indonesia.

The purpose of this study was to examine associations between exposure to NNCC IPC, knowledge and feeding practices among mothers with children under two years of age in rural Indonesia.

## Methods

### Design

This study included an analysis of cross-sectional data collected in rural Indonesia following the 2014–2018 NNCC intervention and represents a collaborative effort between IMA World Health (IMA), the University of Indonesia’s Center for Nutrition and Health Studies, and the Ministry of Health in Indonesia.

### Sample

The study sample consisted of mothers of children under two years of age from three rural districts (Banyuasin, Kubu Raya, and Katingan) located in three provinces (South Sumatera, West Kalimantan, and Central Kalimantan) in Indonesia. Mothers of children under two years of age were targeted because the first 1000 days of life is a critical period for preventing stunting. Rural districts were selected because that is where the risk for stunting is highest. One district was randomly selected from each of the three provinces. A multi-level sampling strategy was used to construct the study sample. Within each of the three rural districts, 30 villages were randomly selected, and each represented a cluster unit. At a more local level, four sub-villages were randomly selected from within each of the 30 villages, in each of the three districts. Finally, five mothers were selected from each sub-village. The target sample size from each of the three districts was 600 mothers, 1800 overall. Exclusion criteria included the inability to speak Bahasa Indonesia and not having a child under two years of age. The final study sample included 1734 mothers.

### Procedure

Ethical approval was obtained from the Ethical Research Committee by the Faculty of Public Health, Indonesia University. Reconstra, a research firm from Jakarta, conducted the data collection. Signed informed consent was sought from each participant prior to the interview and participation of all subjects was voluntary. Survey data were collected using an electronic tablet by experienced interviewers and field coordinators, each of which was trained to use the tablet-based collection system designed to reduce interviewer bias. Each interviewer interviewed approximately six respondents per day in the local language, Basah Indonesia, and reported to field coordinators who then verified the responses and uploaded survey data daily. A data manager checked data and noted any errors. Data cleaning was done prior to analysis.

Posyandu, the community-based health outpost, was the primary site for IPC interventions. From this facility, participating mothers received post-natal care, growth monitoring and health promotion sessions that were held once monthly and focused on: 1) nutrition (maternal, infant, and young child, including breastfeeding and complementary feeding); and 2) sanitation and hygiene (latrine usage and handwashing). Mothers’ participation in IPC programming was completely voluntary.

### Measurement

Demographic information was collected and included the age of the mother and child, the mothers’ education level, and the total household income level, as represented in Indonesian Rupiah. Participation in the IPC intervention was also measured using a yes/no item to reflect participants’ self-reported exposure. Additionally, respondents were asked a series of questions to gauge their knowledge about IYCF. A composite knowledge variable was constructed using participant responses from three variables, including correctly identifying that children should eat at least three meals per day (meal frequency), correctly identifying that children should have at least two snacks per day (snack frequency), and being aware of the benefits of active feeding. Correct responses to each question resulted in a value of 1. The composite variable was the summation of the values for the three questions. This resulted in four categories: *no knowledge*, *low knowledge*, *medium knowledge* and *high knowledge*. The categories of no knowledge and low knowledge were combined because of low representation in each category.

Four variables measuring feeding practices were constructed, consistent with WHO standards for IYCF indicators [1]. These variables included minimum meal frequency (MMF), minimum dietary diversity (MDD), and minimum acceptable diet (MAD). Each are described below. Note that the original IYCF indicator included iron rich foods. Our analysis did not include these because they were not part of the questionnaire.

### Minimum meal frequency

Minimum frequency was defined as at least two meals for breastfed infants 6–8 months, three meals for breastfed children 9–23 months, and four meals for non-breastfed children 6–23 months. Those who met these standards were coded as 1 and if they did not meet the standard, they were coded as 0.

### Minimum dietary diversity

Minimum Dietary Diversity was based off of the IYCF indicator created by the WHO [1]. Mothers were asked to report on all food given to their child in the previous 24 h. If the child consumed at least one food in a given group, he or she was coded as having had that food group. The five food groups considered for dietary diversity included: 1) Grains/staple foods (corn, wheat, rice, grains, roots or tuber), 2) Nuts and beans: peanuts, beans, walnuts, and green beans 3) Vitamin A-rich fruits and vegetables, 4) Animal source foods such as meat, liver, eggs, and fish, and 5) Milk (formula and breastfed). Categories 1, 2, and 5 were coded as 1 point and Categories 3 and 4 were coded as 2 points. The values from each of the five categories were then summed. If respondents scored 4 or above, they met the minimum dietary diversity score (coded as 1). Respondents scoring below 4 did not meet the MDD standard and were coded as 0. Note that due to a limited number of related variables being unavailable, an adapted approach to using the WHO guidelines was used.

### Minimum acceptable diet

Children who met the criteria for MMF and MDD were considered to have achieved a minimum acceptable diet (MAD) and were given a value of 1. Those who met only one or none of these two standards were given a value of 0. This variable was also based on the IYCF variables [1].

### Analysis

SAS software version 9.4 was used to conduct all analyses. Demographic information was analyzed using basic frequency statistics. Descriptive statistics were also used to understand maternal knowledge and the three outcomes of interest (MMF, MDD, and MAD). Both linear and logistic regression analyses were used to describe relationships between exposure to the intervention, knowledge and behavioral practices. Each model controlled for the mother’s age, level of education and total household income as each could impact a mother’s nutrition knowledge or behavior.

## Results

The mean age of mothers responding to the survey was 28.9 years. Information about education level, religion, mean income, and occupation is found in Table 1. A total of 525 (30.3%) mothers reported exposure to IPC. Table 2 contains frequency information about women’s knowledge of feeding practices, after the IPC intervention had already taken place. Most mothers correctly identified recommended meal frequency and snack frequency and were aware of the benefits of active feeding. Half of the sample (52.7%) had a high knowledge of active feeding. Table 3 contains results regarding the percentage of individuals who met the standards for MMF (90.2%), MDD (81.8%), and MAD (78.8%).

**Table 1:** Demographics of the study sample (*n* = 1734)

Education	Number	Percent
Never went to school	97	5.6
Elementary school	670	38.6
Junior high school	423	24.6
Senior high school	434	25.0
College/university/other higher education level	110	6.3
**Religion**		
Islam	1640	94.6
Other	94	5.4
**Occupation**		
Unemployed/housewife	1461	84.3
Daily/industrial worker	13	0.8
Hunter/farmer/fisher	55	3.2
Light traders/shop owner	79	4.6
Other	126	7.3
**Exposure to IPC**		
No	1209	69.7
Yes	525	30.3
	Mean	SD
**Income**^**a**^	2,193,594	1,944,609
Age	28.9	6.3

^a^Indonesian Rupiah

**Table 2:** Nutrition knowledge of Indonesian women included in the study sample

Variable	Frequency	%
**Meal Frequency Knowledge**		
0–2 times a day	264	15.2
3 times a day	1470	84.8
**Snack Frequency Knowledge**		
0–1 times a day	438	25.3
2 times a day	1296	74.7
**Active Feeding Knowledge**		
Yes	483	27.9
No	1251	72.2
**Composite Active Feeding Knowledge**		
No to low knowledge	301	17.4
Medium knowledge	519	29.9
High knowledge	914	52.7

**Table 3:** Frequency of childhood feeding practices of Indonesian women included in the study sample

Dietary Diversity	Frequency	%
Met requirements		
No	219	18.2
Yes	984	81.8
**Meal Frequency**		
Met requirements		
No	111	9.8
Yes	1018	90.2
**Acceptable Diet**		
Met requirements		
No	239	21.2
Yes	890	78.8

Results from linear regression analyses are presented in Table 4. Exposure to IPC was related to increased knowledge of feeding practices (*p* < .0001), after controlling for mother’s age, education, and total household income. Results from logistic regression analyses are presented in Table 5. Increased knowledge was related to increased childhood feeding behaviors, including MMF (*p* = 0.02), MDD (*p* = 0.01), and MAD (p < .0001). Specifically, in unadjusted models, those with a medium level of knowledge regarding IYCF practices overall, were 1.6 times more likely than mothers with little or no knowledge to attain MMF, 1.4 times more likely to achieve MDD, and 1.6 times more likely to attain MAD. In adjusted models, these results were attenuated only slightly. A high level of knowledge was also important. Those with high levels of knowledge, relative to those with (medium levels or no/low levels) were 5.5 times more likely to achieve MMF, 9.2 times more likely to experience MDD, and 5.4 times more likely to attain MAD.

**Table 4:** Association between exposure to IPC and knowledge of feeding practices

	Unadjusted Point Estimate (***P***-value)	Adjusted Point Estimate (***P***-value)
Exposure to IPC	0.2 (<.0001)	0.2 (<.0001)

Note: adjusted model controlled for mother’s age, education, and total household income

**Table 5:** Association between knowledge of feeding practices and childhood feeding practices

Variable	Unadjusted OR (CI)	Adjusted OR (CI)
**Minimum Meal Frequency**		
Medium vs no to low knowledge	1.6 (1.2–2.11)	1.4 (1.1–1.9)
High vs no to low knowledge	2.5 (1.5–4.2)	2.1 (1.2–3.6)
**Minimum Dietary Diversity**		
Medium vs no to low knowledge	1.4 (1.1–1.7)	1.3 (1.1–1.6)
High vs no to low knowledge	1.9 (1.3–2.9)	1.8 (1.2–2.7)
**Minimum Acceptable Diet**		
Medium vs no to low knowledge	1.6 (1.3–1.9)	1.5 (1.2–1.8)
High vs no to low knowledge	2.5 (1.7–3.7)	2.3 (1.5–3.4)

Note: adjusted model controlled for mother’s age, education, and total household income

## Discussion

The purpose of this study was to evaluate the impact of an IPC campaign on childhood feeding knowledge and the extent to which knowledge was then associated with actual childhood feeding behaviors. Knowledge is emphasized as a prominent construct in and determinant of behavior change, fitting into the plausible and theoretical pathway that links exposure to interventions and changes in behavior [17]. In this study, approximately one-third of caregivers were exposed to IPC. Exposure to IPC was associated with increased knowledge of feeding practices and in turn, knowledge was related to actual feeding practices. These were strong and significant findings, even after adjusting for key sociodemographic characteristics. These findings serve to both confirm a theoretical link and also to provide justification for consideration of future IPC interventions in rural Indonesia. MDD, MFF, and MAD are often difficult to change, and these findings provide reasons for optimism. Future research, including implementation research studies using qualitative data collection and program fidelity assessments, should examine the specific program elements that appear to be associated with changes in knowledge and IYCF practices. Additionally, long-term follow-up studies are essential to determining the extent to which changes in behavior relate to downstream changes in stunting in Indonesia.

The value of interpersonal approaches has been demonstrated previously, frequently in the form of lay health advisors or community health workers [18]. A review article of community health workers’ impact on nutritional outcomes confirms that measurable improvements in meal frequency and diet diversity can be achieved in young children [19]. The actual mechanisms by which IPC interventions take effect are not perfectly understood. In some interventions it may actually be a platform for providing and disseminating information. And in others, IPC may be used as a tool to deconstruct messages communicated elsewhere. In this case, IPC may act as a moderator and mediator of other information sources, for example, media [20, 21] and then be a functional agent for behavior change [22, 23] In a study conducted in Nepal, an IPC intervention that involved dialogue groups to discuss information gathered elsewhere and was then effective at shaping attitudes and perceptions about reproductive health topics [24]. Some messages communicated to broad audiences may be in generic, impersonal tones (e.g., a brief television segment) that has an impact on knowledge acquisition, especially with repeated exposures. Then, from within a more comfortable or interpersonal network and from those that have the technical skills, the message is reinforced, which is likely to have a more profound impact on attitudes, beliefs and perceptions [25]. The focus of future research in rural Indonesia should explore the functional behavior change elements of IPC.

Our results indicate that it is possible, with a concerted effort, to reach a large number of individuals at health facilities and that IPC efforts can translate into gains in knowledge, which may then lead to improved IYCF practices. The challenge then becomes sustaining this level of service in rural areas without the support of a research team. Historically, delivering IPC approaches in rural areas is not trivial and IYCF practices can be suboptimal [26]. Moving forward and in the context of Indonesia’s growing use of social media [27], there may be value from an effort to use technology to complement traditional IPC efforts [28]. The notion of community health workers delivering personalized health information via a technology medium has support [29]. It may be worth exploring the feasibility of this type of approach in rural Indonesia.

## Limitations

The results of this study should be interpreted in the context of several limitations. These include the cross-sectional nature of the study, lack of information about stunting status, and lack of indicators that are consistent with WHO guidelines. Lastly, there was just a small number of demographic variables that could be used as control variables in the multivariate models. This was due to some potential demographic variables being dominated by single categories. Future studies may benefit from the inclusion of additional control variables including things such as social norms, cultural practices and the inclusion of spouse data, which were not available in this study. Strengths include a large sample size, randomization of sampling units, and the presence of a large-scale intervention.

## Conclusions

The findings from this study are important as they provide support for a large-scale intervention to address knowledge among rural mothers. Future efforts in rural Indonesia may take note of the associations presented here and build upon these findings to expand programs and services.

## Data Availability

The data that support the findings of this study are available from IMA World Health but restrictions apply to the availability of these data, which were used under license for the current study, and so are not publicly available. Data are however available from the authors upon reasonable request and with permission of IMA World Health.

## Abbreviations

NNCC: National Nutrition Communication Campaign
IPC: Interpersonal Communication
MMF: Minimum Meal Frequency
MAD: Minimum Acceptable Diet
MDD: Minimum Diet Diversity
IYCF: Infant and Young Child Feeding
